# Possible Single-Nucleotide Polymorphism Loci Associated with Systemic Sclerosis Susceptibility: A Genetic Association Study in a Chinese Han Population

**DOI:** 10.1371/journal.pone.0113197

**Published:** 2014-12-03

**Authors:** Chang Shu, Wei Du, Xiaofei Mao, Yun Li, Qin Zhu, Wei Wang, Nan Wu, Xuming Mao, Hongzhong Jin, Qiuning Sun

**Affiliations:** 1 Department of dermatology, Peking Union Medical College Hospital, Chinese Academy of Medical Sciences, Beijing (100005), China; 2 Department of surgery, Peking Union Medical College Hospital, Chinese Academy of Medical Sciences, Beijing (100005), China; 3 Department of Dermatology, University of Pennsylvania, Philadelphia, Pennsylvania, United States of America; University of Texas Health Science Center at Houston, United States of America

## Abstract

**Objective:**

The aim of this study was to confirm the association of *RHOB* and *FAM167A-BLK* gene polymorphisms with susceptibility to systemic sclerosis (SSc) in a Chinese Han population.

**Methods:**

A total of 248 SSc patients and 251 healthy controls of Chinese Han ethnicity, which visited the department of dermatology of Peking Union Medical College Hospital, were included in the study. Six selected single nucleotide polymorphisms (SNPs) in the *RHOB* and *FAM167A-BLK* regions were selected as markers and were genotyped using a MassARRAY system, which is based on the matrix-assisted laser desorption/ionization time of flight mass spectrometry technique.

**Results:**

Three SNPs in the coding regions of the *RHOB* and *FAM167A-BLK* genes displayed an association with SSc: (1) rs1062292T, which is a newly discovered SNP in the *RHOB* gene (P = 0.03, odds ratio [OR] = 1.62, 95% confidence interval (CI) = 1.05–2.50), (2) rs2736340T (P = 0.03, OR = 1.39, 95%CI = 1.03–1.85), and (3) rs13277113A (P = 0.04, OR = 1.34, 95%CI = 1.01–1.76), both in the *FAM167A-BLK* gene. Our results support previous findings that vaiants in the *RHOB* and *FAM167A-BLK* genes may be associated with susceptibility to SSc. However, the loci of the SNPs in *RHOB* region that displayed an association with SSc are quite different from the loci which were identified in studies of Caucasian populations.

**Conclusion:**

Our results confirm that *RHOB* and *FAM167A-BLK* polymorphisms exist in Chinese Han SSc patients. Therefore, variants of the *RHOB* and *FAM167A-BLK* genes are promising genetic markers for SSc.

## Introduction

Scleroderma or systemic sclerosis (SSc) is a chronic, connective tissue disease characterized by widespread fibrosis of the skin and internal organs, small-vessel vasculopathy, and immune dysregulation with or without production of autoantibodies. SSc patients have markedly shorter life span than that of the age- and sex-matched general population. In a recently published meta-analysis, the overall pooled standardized mortality ratio of patients with SSc was 3.53 [1]. During the past few years, knowledge of the genetic basis of SSc has increased rapidly because of large and well-powered candidate gene association studies [2], [3] as well as genome-wide association studies (GWASs) [4]–[6]. Currently, it is widely accepted that different genetic factors contribute to the development and prognosis of SSc. Further, GWASs have been a useful tool for studying the genetic basis of autoimmune and other complex diseases. Radstake *et al.* [4] performed the first SSc GWAS in a Caucasian population, which also represented the first large-scale GWAS in an SSc cohort. In a recent GWAS in a French Caucasian SSc discovery cohort [5] 17 single-nucleotide polymorphisms (SNPs) displaying tier two associations were selected for follow-up in independent cohorts. Three of the selected SNPs were located within the human leukocyte antigen (HLA) region corresponding to the *HLA-DQB1* and *PSORS1C1* genes. The remaining SNPs were located in six independent non-HLA loci. After the replication step, six SNPs located in three loci (*TNIP1, RHOB*, and *PSORS1C1*) were proposed as novel SSc risk factors. However, later, in a large independent replication study by a Spanish group, *TNIP1*, but not *RHOB* and *PSORS1C1*, was confirmed to be associated with SSc [7].

The associations identified in a single GWAS, despite crossing established statistical significance thresholds, tend to display inflated effect sizes. This effect size is called the winner's curse, and it affects the predictive ability of the discovered associations and the estimation of the risk variance based on the associations. Thus, it is essential to replicate these studies in independent comparable populations for firmly establishing a genotype-phenotype association. On the other hand, China has a large SSc patient population, and genotyping data is lacking for this population. Peking Union Medical College Hospital (PUMCH) is believed to have the largest SSc patient group in China, and a large portion of this group regularly visits the dermatology department. We collected SSc data for more than three years, including data on two major subgroups: limited (lcSSc) and diffuse systemic sclerosis (dcSSc), the latter of which has the worse prognosis. Therefore, we performed a replication study in a previously unexamined SSc population to confirm the results of previous GWAS and candidate gene association studies.

*RHOB* is the ras homolog gene family member B that regulates protein signaling and intracellular protein trafficking. It was first reported in a GWAS study, but signal of association was weaker at this locus [5]. Replication denied that *RHOB* was associated with SSc in Caucasian population [7]. We were wondering if such association could be examined in another ethnic group such as Chinese population. Also as a small gene, only several tag-SNPs would be chosen, and this would be easier for us to start such an exploration with a limited expense.

*FAM167A* (family with sequence similarity 167 member A) and *BLK* (B lymphoid tyrosine kinase) also known as *C8orf13-BLK*, was identified as a susceptibility locus for SSc and analyzed in different SSc cohorts. In the first report of BLK influence in SSc genetic predisposition, the combined analysis of Caucasian US and European cohorts revealed the association of the minor allele of two genetic variants (rs13277113 and rs2736340) with increased susceptibility to SSc, and specifically with the lcSSc and Anti-centromere antibody (ACA) positive subsets [3]. Analysis of rs13277113 polymorphism in a Japanese cohort showed association of this marker with the whole disease independently of the subtype or autoantibody subgroup [2]. The differences of minor allele frequency (MAF) in the two loci between the two ethnic groups drove us to put the two loci into our study.

## Patients and Methods

### Subjects

This study was approved by the Ethics Committee of Peking Union Medical College Hospital. Both patients and controls were included in the study after providing written informed consent. DNA was obtained from patients and controls using standard methods.

We performed a case-control association study in 248 SSc patients from the outpatient dermatology and rheumatology departments at the PUMCH. Two hundred and fifty-two controls were recruited from the physical examination center of PUMCH. All subjects were of Chinese Han ethnicity and from the mainland of China. All the patients fulfilled the 1980 American College of Rheumatology classification criteria for SSc. Among the 248 patients, 109 (44%) were dcSSc and 139(56%) were lcSSc. The control population consisted of unrelated healthy individuals who were from the same geographical regions as the SSc patients and who were matched by age group and sex with the SSc patients, as shown in Table 1.

**Table 1:** Demographic information of the patients and controls.

Features	SSc patients	Healthy controls
Patients	248	252
Age <18	10	10
Age 18–44	131	133
Age 45–59	83	85
Age >60	24	24
Average age (year)	42.7	42.7
Age range (year)	14–81	20–77
Male (%)	11.3	11.1
Diffuse (%)	44.0	-

### Genotyping

The rs1062292, rs13021401, rs342054, and rs342070 alleles were selected as genetic markers of *RHOB*. rs342070 and rs13021401 were previously identified in a GWAS [5], and the other two were selected via tagger SNP analysis implemented in HaploView 4.2 [8] at an r^2^ threshold of 0.80 and an LOD threshold for multi-marker tests of 3.0. rs2736340 and rs13277113 in *FAM167A-BLK* have been previously reported to be associated with SSc in a Japanese population [2], [3]; therefore, we decided to confirm whether the same association exists in a Chinese population.

Genomic DNA was extracted from the peripheral blood of each subject. The six SNPs were genotyped using a matrix-assisted laser desorption/ionization time of flight mass spectrometry genotyping assay using the MassARRAY platform from Sequenome following the manufacturer's suggestions (Foster City, CA, USA). The genotyping call rate was larger than 95% for all six SNPs.

### Statistical analysis

SHEsis [9] was used for the individual population association tests (significance was calculated using 2×2 contingency tables and Fisher's exact test or a χ^2^-test when necessary).SHEsis is a web-based platform for analyses of linkage disequilibrium, haplotype construction, and genetic association at polymorphic loci designed by YongYong Shi from Shanghai Jiao Tong University. The software calculations are based on an expectation-maximization algorithm [9], [10]. The odds ratios (OR) and their 95% confidence intervals (CI) were also reported by the software. All cohorts were in Hardy–Weinberg equilibrium (HWE) at a significance level of 0.05 for all the included SNPs. Power was calculated using the software Power Calculator for Genetic Studies 2006 and assuming an additive model at the 5% significance level. For *RHOB*, with an OR of 1.5, taking into account the expected frequency of the rare allele of rs1062292, the set has a power of 78%. Similarly, for rs13021401, the set has a power of 78%; for rs342054, the set has a power of 75%; for rs342070, the set has a power of 79%. For *FAM167A-BLK*, with an OR of 1.5, the set for rs2736340 has a power of 79%, and the set for rs13277113 has a power of 79%.

## Results

### Frequencies of Alleles and Genotypes in Patients and Controls

The genotype distribution of the six SNPs was in HWE in the controls (P>0.05). The results of the association are shown in Table 2. As the data indicate, the rs1062292T allele showed a higher prevalence in SSc patients (91.9%) than in controls (87.6%), thereby indicating a statistically significant association between the rs1062292T allele and SSc risk (P = 0.03, OR = 1.62, 95%CI = 1.05–2.50) in the χ2 test. The rs2736340T allele was associated with higher risk of SSc in patients (74.9%) than in controls (68.3%, P = 0.03, OR = 1.39, 95%CI = 1.03–1.85). Similarly, the rs13277113A allele was associated with higher risk of SSc in patients (72.6%) than in controls (66.5%, P = 0.04, OR = 1.34, 95%CI = 1.01–1.76). No significant differences were observed between the SSc patients and controls for the other SNPs. For the limited sample size and small number of SNPs which were tested, also the power is also not as high as a usual study requested, no further correction for multiple testing has been performed, and therefore less false negative would not be dropped. That is the reason of unadjusted P-value is presented here. Linkage disequilibrium analysis was also made among SNPs that were tested in the *RHOB* region using HaploView 4.2. Between rs1062292 found here and both two SNPs previoiusly reported by Allanore *et al.* showed no significant linkage (D′ = 0.31, r^2^ = 0.03).

**Table 2:** Frequencies of Alleles in Patients and Controls.

Gene region	SNP	Minor Allele	Cases n = 238	Controls n = 245	*P* value	OR (95%CI)
*RHOB*	rs1062292	G	37(0.081)	57(0.124)	**0.03**	**T: 1.62 (1.05–2.50)**
*RHOB*	rs13021401	C	162(0.352)	174(0.378)	0.41	
*RHOB*	rs342054	T	23(0.049)	25(0.052)	0.86	
*RHOB*	rs342070	A	178(0.376)	187(0.383)	0.81	
*FAM167A-BLK*	rs2736340	C	111(0.251)	144(0.317)	**0.03**	**T: 1.39 (1.03–1.85)**
*FAM167A-BLK*	rs13277113	G	130(0.274)	163(0.335)	**0.04**	**A: 1.34 (1.01–1.76)**

## Discussion

Allanore *et al.* identified *RHOB* to be associated with SSc susceptibility in a GWAS aimed at identifying loci associated with SSc risk. [5] We confirmed the association of *RHOB* with SSc, but the loci identified are slightly different from the ones identified by Allanore *et al*. However, in a replication study of the same GWAS in Caucasians [7], association between *RHOB* and SSc was not observed. This interesting contradiction may be explained by the genetic heterogeneity among human races. rs1062292, which displayed an association with SSc in our study, is an SNP located in the 3′-untranslated region (3′-UTR) of *RHOB*. This region is commonly involved in post-transcriptional regulation. The exact roles that this SNP plays require further exploration. The RhoB protein mediates apoptosis in neoplastically-transformed cells after DNA damage [11]. *RHOB* is not essential for development but affects cell adhesion and growth factor signaling in transformed cells [12].This gene is also required for stability and nuclear trafficking of AKT1/AKT, which promote endothelial cell survival during vascular development and may play a role in SSc pathogenesis [13]. The SNP identified in this study has not been reported previously. It appears that the polymorphism at this locus is distinctive in Chinese SSc patients. Concerning the limited sample size of this study, larger case-control group and denser SNP genotyping are required before association with SSc can be definitely made.

*FAM167A* (previously referred to as *C8orf13*)-*BLK* is a region in chromosome 8. *BLK* encodes a nonreceptor tyrosine kinase of the src family of proto-oncogenes, which are typically involved in cell proliferation and differentiation. The protein plays a role in B-cell receptor signaling and B-cell development [14], [15].

The association of SNPs in the *FAM167A-BLK* region with systemic lupus erythematosus (SLE) has been demonstrated in Caucasians and Asians. The SNP in this region was first found to be associated with SLE through a GWAS [16]. Previous findings indicate that the rs2736340C and rs13277113A alleles are associated not only with SLE but also with SSc in European and Japanese populations [2], [3] and that the SNP polymorphisms of *FAM167A-BLK* region are a common genetic risk factor for both SLE and SSc. The association of these two SNPs with SSc was also confirmed in our study. It was also confirmed in this study that both the allele frequency for rs2736340T and rs13277113A was 74.9% and 72.6% in cases, and 68.3% and 66.5% in controls respectively, indicating that the high LD between the two associated SNPs located near the 5′ ends of *FAM167A* and *BLK*. The similar result has been reported by Gourh P et al. Interestingly, rs2736340T and rs13277113A which identified as the minor allele in Caucasians are the major allele in Asians but give similar strengths of associations. In addition, polymorphisms in this region have been determined to be associated with other immune diseases such as Kawasaki disease, Sjögren's syndrome, and rheumatoid arthritis (RA) [17]–[19]. These results suggest that the B-cell receptor signaling pathway may play an important role in the pathogenesis of multiple immune diseases.

Other genes such as STAT4 and loci in HLA regions which associated with SSc in Caucasian and Japanese population were also confirmed in Chinese population [20], [21]. The genes that associated with SSc are mostly involved in immune regulation and inflammation. Many of these genes also have been found to be associated with other autoimmune diseases such as SLE and RA [22]. The use of the new genetic technologies such as immunochip might be more efficient and replicable to fully characterize the genetic component of SSc in a new ethnic group[23]. Hopefully, genetic screening of the patients might provide valuable help in understanding this complex disease.

## Conclusion

Our results confirm that *RHOB* and *FAM167A-BLK* polymorphisms exist in Chinese Han SSc patients, which indicates that *RHOB* and *FAM167A-BLK* variants may be associated with susceptibility to SSc. However, the SNPs in *RHOB* region that displayed an association with SSc are quite different from those in Caucasian populations. Moreover, we observed a new SNP, rs1062292T, which was significantly associated with the higher risk of SSc in Chinese Han populations. This information may help us to identify future molecular targets for disease diagnosis and therapy.
